# Are predictive saccades linked to the processing of peripheral information?

**DOI:** 10.1007/s00426-022-01743-2

**Published:** 2022-09-27

**Authors:** Christian Vater, David L. Mann

**Affiliations:** 1 Institute of Sport Science, University of Bern, Bremgartenstrasse 145, 3012 Bern, Switzerland; 2 Faculty of Behavioural and Movement Sciences, Motor Learning and Performance, Vrije Universiteit Amsterdam, Amsterdam, The Netherlands

## Abstract

High-level athletes can predict the actions of an opposing player. Interestingly, such predictions are also reflected by the athlete’s gaze behavior. In cricket, for example, players first pursue the ball with their eyes before they very often initiate two predictive saccades: one to the predicted ball-bounce point and a second to the predicted ball-bat-contact point. That means, they move their eyes ahead of the ball and “wait” for the ball at the new fixation location, potentially using their peripheral vision to update information about the ball’s trajectory. In this study, we investigated whether predictive saccades are linked to the processing of information in peripheral vision and if predictive saccades are superior to continuously following the ball with foveal vision using smooth-pursuit eye-movements (SPEMs). In the first two experiments, we evoked the typical eye-movements observed in cricket and showed that the information gathered during SPEMs is sufficient to predict when the moving object will hit the target location and that (additional) peripheral monitoring of the object does not help to improve performance. In a third experiment, we show that it could actually be beneficial to use SPEMs rather than predictive saccades to improve performance. Thus, predictive saccades ahead of a target are unlikely to be performed to enhance the peripheral monitoring of target.

## Introduction

In daily life, we often predict the consequences of our actions, which is best visible in situations where the expected consequence does not match the actual outcome. For example, when stairs are not as high as we predict, we tumble because the foot was not lifted high enough (Marigold et al., 2007). With greater task experience, predictions become more accurate (Vater et al., 2022). In sports, elite athletes can predict the actions of an opposing player very reliably (Loffing & Cañal-Bruland, 2017). In tennis, for example, prediction is particularly required under time pressure when both players are close to the net. In this situation, it would be difficult to reach the ball when reacting after the opponent hits the ball (Triolet et al., 2013). Instead, players predict the likely shot direction based on visual cues about the posture of the opponent or knowledge about the player’s preferred shot direction (Abernethy et al., 2001; Triolet et al., 2013). Interestingly, these predictions are also reflected by the athlete’s gaze behavior. For example, in baseball, cricket, table tennis, and squash, predictive eye-movements are made to future location(s) along the ball’s trajectory (Bahill & LaRitz, 1984; Hayhoe et al., 2012; Higuchi et al., 2018; Land & Furneaux, 1997; Land & McLeod, 2000; Ripoll, 1989; Rodrigues et al., 2002). If the ball bounces, these predictive saccades are generally initiated to the future bounce point. Yet, the functionality of these predictive saccades is still unclear (Diaz et al., 2013; Mann et al., 2013). Clearly, immediately after every predictive saccade, the relevant target object, in these cases the ball, is positioned in the peripheral visual field of the athlete. The goal of the present series of studies is to determine how functional predictive saccades are for processing peripheral information. Since gaze behavior in these situations consists largely of a pursuit eye-movement, followed by a (predictive) saccade, and a fixation after this saccade, we will compare motion perception during pursuit, fixation, and finally, in two ‘eye-cricket’ experiments, simulate the predictive saccades, with a combination of pursuit, saccade and fixation.

Cricket provides an ideal environment in which to understand predictive eye-movements. In cricket, a bowler bowls the ball in the direction of a batter. After the ball leaves the hand of the bowler, it bounces 2–10 m from the batter. The goal of a batter is then to hit the ball with their bat. The ball is released by a bowler with speeds up to 160 km/h (Croft et al., 2008; Müller et al., 2009), and the batter can have less than 600 ms from ball release to arrival (Sarpeshkar & Mann, 2011). Results by Renshaw and Fairweather (2000) indicate that expert cricket players can use information from the postural cues of the bowler to discriminate different ball types, even before the ball is released. Müller et al. (2009) showed that the ball-flight information between ball release and bounce is particularly important for batters to successfully intercept the ball after the bounce. Since expert cricket batsman only follow the ball for the first 100–150 ms of ball flight (Land & McLeod, 2000), they seem to pick up the relevant characteristics of the ball flight (i.e., speed and trajectory) very quickly.

During the ball flight when batting, players often initiate two predictive saccades: a first to the predicted point of ball-bounce, and a second to the predicted bat-ball contact point. The first saccade occurs after tracking the ball for the first portion of ball flight, around 100–200 ms ahead of the actual bounce depending on the ball speed (Land & McLeod, 2000). Interestingly, Land and McLeod (2000), who investigated three players with different cricket expertise, found that a highly skilled player initiated their saccade earlier than the batters with lower skill level. This result, however, could not be replicated in a study with a higher number of high and low skilled players (Sarpeshkar et al., 2017). The second predictive saccade is sometimes initiated to the place where the bat will make contact with the ball. This predictive saccade might be used because, as the ball is closer to the batsman, the large changes in viewing angles might make it difficult to use pursuit eye movements (Mann et al., 2019). In one study, it has been reported that batters do not or cannot watch the ball when it is hit (Land & McLeod, 2000) yet a subsequent study has shown that they can by using the second predictive saccade (Mann et al., 2013).

Mann et al. (2013) proposed three potential functionalities for predictive saccades. First, predictive saccades may facilitate ball tracking after the ball bounce to a degree that is better than what would be possible when tracking the ball. The ball at bounce undergoes a considerable change in the angular velocity apparent at the eye, and it may be difficult for observers to pursue smoothly through this discontinuity in the motion path. Batsmen could anchor their gaze near the bounce location to avoid this discontinuity and facilitate tracking after the bounce (Mann et al., 2013, p. 10, see also Brenner & Smeets, 2011; Diaz et al., 2013; Hayhoe et al., 2005; Mann et al., 2013). Second, batters make predictions about where the ball will bounce and arrive, and predictive saccades to bounce could serve a purpose to check whether the predicted location of bounce aligns with the actual location of bounce in order to provide an accurate form of feedback for future predictions (Mann et al., 2013, p. 10). Third, predictive saccades may aid “batters to better detect, and subsequently adapt to, unexpected changes in the flight-path of the ball after it bounces” for instance when bouncing off an irregular or rough surface (Mann et al., 2013, p. 10).

It is possible that after predictive saccades, peripheral vision is used to make fast adjustments to the visually guided hitting action (Vater et al., 2020b). For instance, despite not tracking the ball during the final phase of the ball flight after bounce, some cricket players have reported that they make late visually guided adjustments to their movements, for instance by adjusting their wrist orientation in the final phase hitting the ball (Mann et al., 2013). If true, the batters must presumably be using their peripheral vision because they did not directly look at the ball. Similarly, in table tennis, elite athletes “anchor” their gaze at the expected hitting location and seem to use peripheral information to adjust their bat swing (Bootsma & van Wieringen, 1990). This finding supports the assumption that athletes process ball information after the predictive saccade, i.e., when the ball is in their peripheral vision. Similarly in a racquetball study, it was reported that predictive saccades toward bounce land slightly above (rather than on) the predicted ball-bounce location and are initiated 300–400 ms before the ball actually reaches the bounce (Diaz et al., 2013). Since the updating of visual information takes only 80 ms (Diaz et al., 2013), it is likely that information was updated using peripheral vision.

As could be seen so far, a typical gaze pattern in hitting sports consists of smooth pursuit eye movements (SPEMs), and saccades with subsequent fixations. When looking at fundamental research on SPEMs, humans are very accurate in following objects with their eyes and can adjust their eye-movement velocity to different speed perturbations (Gegenfurtner et al., 2003). A similar accuracy is observed in psychometric performance (i.e., the ability to detect the perturbation, Braun et al., 2010; Gegenfurtner et al., 2003). In another study, it has been shown that short periods of pursuit tracking (between 100 and 300 ms) enable the prediction of whether an object will hit a target or not (Fooken et al., 2016). In their study, participants (baseball players) had to track a virtual ball with their eyes and saw only the initial 100 ms, 200 ms, or 300 ms of its trajectory, after which participants were required to anticipate, using their index finger, when the ball would have hit a target zone. The results showed that more accurate SPEM tracking led to better predictions. That is, participants successfully used retinal information obtained during their SPEMs and presumably also extraretinal signals to predict the future location of the target (for a review see Fooken et al., 2021). However, the duration of the initial target presentation period did not alter hitting accuracy. It could be that a combination of SPEMs and a saccade (as in sports) would be more beneficial than using pursuit alone when it comes to hitting accuracy.

After tracking the ball with the eyes, a batter’s eye-movements are characterized by a saccade ahead of a target. Basic research shows that saccades are pre-programmed and that visual attention is covertly shifted to the target of the saccade (for a review on eye-movements and selective attention see Souto & Kerzel, 2021). Saccades can also impair or even suppress information processing (for a review see Binda & Morrone, 2018). Observers are, for example, very poor in detecting image displacements during saccades (Bridgeman & Stark, 1991), or have distorted perception shortly before the onset of a saccade (Ross et al., 1997). It is summarized that saccades (a) suppress visual sensitivity and dampen the sensation of motion, and (b) lead to a “gross perceptual distortion of visual space in anticipation of the repositioning of gaze” (Ross et al., 2001, p. 113). If motion perception is impaired during saccades, it is unlikely that information processing occurs during the predictive saccade. Instead, batsman probably uses their peripheral vision to process visual information during the fixation that occurs after the predictive saccade.

This leads us to the third area of relevant basic research: the processing of motion in peripheral vision. When viewing motion changes with peripheral vision during fixation, motion changes can be detected across different viewing eccentricities and the time to detect these changes is independent of eccentricity—in contrast to SPEMs, where larger eccentricities lead to prolonged detection times (Vater et al., 2020a, 2020b). Motion speed, however, is typically underestimated during fixation when using peripheral vision, and the magnitude of underestimation increases with eccentricity (Traschütz et al., 2012). This result holds irrespective of the speed and contrast of stimuli (Hassan et al., 2016). Thus, a moving ball viewed in peripheral vision should be perceived to be slower than it actually is. That means, when time-to-contact (TTC) is crucial, as it is the case when hitting a ball in cricket, TTC could be impaired because the ball is perceived slower than it actually is. Interestingly, research on the Aubert–Fleischl illusion (Aubert, 1886; Fleischl, 1882) found that an object appears to move slower when it is pursued than when it is viewed during fixation, leading to the opposite prediction (Fleischl, 1882).

When returning to the predicted functionalities of saccades by Mann et al. (2013), and considering the reviewed literature, it may be that it is not the predictive saccade per se that facilitates or aids performance, but rather the fixation thereafter. Since the ball is viewed in peripheral vision during that fixation, peripheral monitoring could explain the use of predictive saccades. In a related study, Spering et al. (2011) tested if the motion direction of a moving target is better predicted when using SPEM or fixation (using peripheral vision) and found better performance in the SPEM condition (for similar results see Bennett et al., 2010; Brenner & Smeets, 2010; van Donkelaar & Lee, 1994). Our aim of the study is now to simulate predictive saccades, with a combination of pursuit, saccade and fixation. If saccades with subsequent fixations ahead of a target would indeed facilitate peripheral monitoring, better speed-change detection rates and TTC estimations should be found compared with SPEM alone conditions. Both of these hypotheses will be tested in this study. In the first two experiments, a SPEM of a moving target will be followed by a saccade and a subsequent fixation ahead of the target. To indicate a perceived speed change, participants were instructed to initiate a saccade to a fixation “+” and continue monitoring the target with peripheral vision and predict when the target would completely overlap with the “+” (TTC). In these two experiments, target occlusion times were systematically varied (Experiment 1: 1000 ms and 1500 ms; Experiment 2: 300 ms, 500 ms, 700 ms, 900 ms) to test whether longer peripheral monitoring times improve TTC performance. If peripheral vision is indeed used for target monitoring, better TTC estimations should be found when the target is occluded later (i.e., with longer monitoring time). In the third experiment (control experiment), participants will use SPEMs only and indicate the speed-change detection with a button press instead of a saccade. This allows us to directly compare the costs associated with predictive saccades in combination with peripheral monitoring. If saccades impair motion processing, better TTC estimations should be found in Experiment 3 compared to Experiment 2.

## Experiment 1

In the first experiment, we will simulate the typical cricket-gaze behavior where participants first pursue the target, then initiate a saccade ahead of the target to a pre-defined location and use their peripheral vision for target monitoring. If peripheral vision is used for monitoring, participants should become better at estimating when the target would align with the fixation point with more peripheral monitoring time. Two advantages arise from a longer monitoring time: first, target information can be processed for a longer period of time and, second, eccentricity declines over time. To manipulate peripheral monitoring time, the target will be occluded either “early” or “late” after a target-speed change. In cricket, it could be expected that a saccade ahead of the target is initiated after the crucial target information is gathered. Similarly, in this experiment, participants will be asked to initiate a saccade after they perceived a target-speed perturbation (i.e., the crucial information for the task). When they perceived no speed change, they should continue pursuing the target. We predicted that there would be a speed effect for the percentage of correctly identified speed perturbations, with best performance in the 3°/s trials. The percentage correct as well as saccadic reaction times should be similar for both occlusion conditions, as participants were asked to initiate a saccade after they detected a target-speed change, and those changes all occurred before the moment of occlusion. TTC predictions should be better in the long monitoring condition because of the availability of additional information.

### Method

#### Participants

Participants were naive to the study aims and self-reported normal or corrected-to-normal visual acuity. The experimental procedures were approved by the faculty ethics committee. All participants provided written informed consent before the experiments. For power calculations, a large effect size ($\hat{\eta }_{{\text{p}}}^{2} = 0.33$) was expected based on a previous study on peripheral motion perception during fixation (Vater et al., 2020a, 2020b). Fourteen student participants (7 female, 7 male, age: *M* = 20.09 years) took part in Experiment 1.

#### Stimuli

**Fig. 1:**
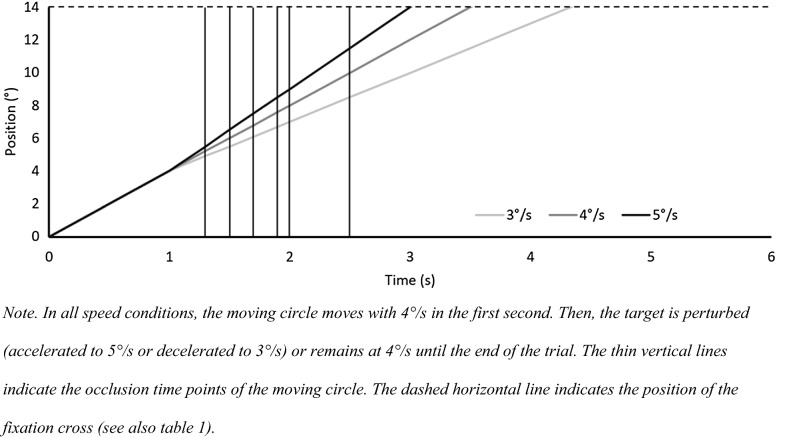
Space–time Plot of the Stimulus

Stimuli were presented on a large back-projected screen (height: 1.87 m; width: 3.01 m; projector: InFocus IN5110, Portland, USA) located 1.2 m from the participant showing a 1.4 m × 1.4 m square (corresponding to 60° × 60°of visual angle). At the beginning of each trial, one white circle (radius = 20 mm; i.e., 2° of visual angle) and one white “+” sign (width = 40 mm; height = 40 mm; corresponding to 4° × 4° of visual angle) appeared on the black screen. The circle was positioned at the same height on the right or left side of the “+”. After 1 s, the circle started to move in the direction of the plus at a speed of 4°/s. After 1 s, the circle decreased speed to 3°/s, increased speed to 5°/s or continued to move with a constant speed of 4°/s (speed condition was the first independent variable). The target was occluded 1 s or 1.5 s after the speed change (occlusion time was the second independent variable) but continued to move at 3°/s, 4°/s or 5°/s, depending on the speed condition (Fig. 1). We expected participants to initiate a saccade within the first 500 ms after perturbation onset so that they would have a relatively short (around 500 ms in the 1 s occlusion interval) or relatively long (around 1000 ms in the 1.5 s occlusion interval) time to monitor the target with their peripheral vision. The eccentricity between the circle and the plus was 14^o^ at the beginning of the trial and 10° at the moment of speed-change onset. Please see Table 1 for an overview of speed conditions, occlusion times, TTC times and peripheral viewing times. Trajectory data were exported as.trc-files and imported to Autodesk 3ds Max (2018) to render single video trials. We presented 40 trials for each combination of speed and occlusion, resulting in 240 trials in total. The trial order was quasi-randomized, ensuring that the same speed-change condition did not occur more than three times in a row.

**Table 1:** Temporal and spatial characteristics of the stimuli

Speed	Occlusion time after perturbation (ms)	Eccentricity from fixation cross at occlusion (in °)	Time to TTC (in s)	Approx. peripheral monitoring time (in s)
3	300	9.1	3.03	0
	500	8.5	2.83	0.2
	700	7.9	2.63	0.4
	900	7.3	2.43	0.6
	1000	7	2.33	0.7
	1500	5.5	1.83	1.2
4	300	8.8	2.20	0
	500	8	2.00	0.2
	700	7.2	1.80	0.4
	900	6.4	1.60	0.6
	1000	6	1.50	0.7
	1500	4	1.00	1.2
5	300	8.5	1.70	0
	500	7.5	1.50	0.2
	700	6.5	1.30	0.4
	900	5.5	1.10	0.6
	1000	5	1.00	0.7
	1500	2.5	0.50	1.2

Occlusion times 1000 ms and 1500 ms were used in Experiment 1. The other four occlusion times were used in Experiment 2 and 3. To calculate peripheral monitoring time, it was assumed that a saccade in response to a perturbation would be initiated after around 300 ms, which was confirmed in Experiment 1 and 2. Thus, if the target occlusion occurs 300 ms after perturbation and the participant initiates a saccade to the + 300 ms after the perturbation, peripheral monitoring time is 0 because the target is occluded when the saccade lands on the +

#### Apparatus

Gaze data were recorded using an integrated binocular eye-tracking system (EyeSeeCam, ESC, 220 Hz, EyeSeeTec GmbH, München, Germany), which assesses the vertical and horizontal rotations of both eyes via infrared reflections from the pupil and the cornea (measurement accuracy: 0.5° of visual angle with a resolution of 0.01° RMS within 25° of the participant’s field of view). The ESC was connected to a MacBook Pro via a 20 m fiber-optic Fire Wire cable (GOF-Repeater 800, Unibrain). Three retro-reflective markers were attached to the ESC and tracked using a 10-camera VICON-T20 system (200 Hz, Vicon Oxford, Oxford, UK) that allowed the calculation of a three-dimensional gaze vector, updated every 5 ms (Kredel et al., 2015). Participants’ responses were recorded in writing (Experiment 1a) or with a Wii remote controller (Nintendo, Kyoto, Japan) that was integrated into the VICON data collection system and was synchronized with the gaze data (Experiment 1b). A naive student assistant controlled the experimental procedure.

#### Procedure

After signing informed consent, participants were fitted with the ESC and positioned 1.2 m from the screen to read the task instructions. Their task was to follow the circle with their eyes (pursuit), initiate a saccade to the plus as soon as the target speed changes, continue monitoring the target with peripheral vision and press the A-button of the Wii as soon as the target (circle) completely overlaps with the plus. After five familiarization trials, the ESC was calibrated. Re-calibration took place after each block of trials (15 trials), and whenever the point of gaze deviated by more than 0.5° of visual angle from one of the dots on a calibration grid projected on the screen. The experiment lasted between 90 and 120 min per participant. Afterward, participants were thanked and debriefed.

#### Analyses

The eye-position signal was differentiated to obtain an eye-velocity profile, which was then smoothed using a 60 Hz low-pass filter. A velocity-based saccade-detection algorithm (Nyström & Holmqvist, 2010) with a fixed velocity threshold of 22°/s (cf., Schütz et al., 2007) was used. When a saccade was detected, periods of 16 ms before and 70 ms after the saccade were excluded before calculating pursuit-eye velocities (c.f., Gegenfurtner et al., 2003). Furthermore, the distance between the two-dimensional gaze location on the screen and the two-dimensional location of the circle or the “+” sign were calculated. In pursuit and fixation trials, the distance between the circle or “+” sign should not exceed 3° of visual angle—a threshold used for peripheral motion detection in previous research (e.g., Vater et al., 2017a, 2017b). Trials were excluded from the analyses if the distance between the gaze location and the circle (pursuit phase) or the plus sign (fixation phase) exceeded the 3°-distance threshold. Participants were excluded from the analyses if they had less than 10 valid trials in a factor combination.

As a first dependent variable, we calculated the percentage of trials with a correct response. A correct response was defined as a trial where a saccade to the cross was initiated when there was a perturbation, and no saccade was initiated (only SPEMs) in trials with no perturbation. The number of correct trials was multiplied by 100 and divided by the number of valid trials (trials with valid gaze data, see above) in the respective condition. Second, we determined the saccadic response time relative to the target’s speed-change onset (in ms). Third, we calculated the prediction error measured as the time difference between the actual TTC and the participants’ predicted TTC (button press, in ms). For this analysis, we only included trials with correct initial perceptual judgements. Because the target speed remained at 3°/s, 4°/s or 5°/s, depending on the speed condition until the end of the trial, the time between occlusion and TTC was different between the speed conditions (see Table 1). Dependent variables were analyzed using ANOVA testing. We expected best response accuracies (percentage correct) for the 3°/s condition compared to the other two speed conditions, and that the relative prediction error should be smaller in the 1500 ms-occlusion condition compared with the 1000 ms-occlusion condition.

The “afex”-package (Singmann et al., 2020) was used for repeated-measures ANOVAs. A two-way ANOVA with the factors speed (3°/s, 4°/s, and 5°/s) and occlusion (1000 ms and 1500 ms) was used to compare response accuracies, saccadic response times and TTC predictions. The “emmeans”-package (Russell, 2019) was used for post hoc contrasts. *p* values were adjusted with the Holm-method. In case the sphericity assumption was violated, Greenhouse–Geisser corrections were applied. Post hoc comparisons were Bonferroni adjusted. Effect sizes were calculated as partial eta squares. For plotting the data, we used the “papaja”—(Aust & Barth, 2020), “bookdown”—(Xie, 2020) and “rmarkdown”—packages (Allaire et al., 2020). Plots show the median of all participants and 25th and 75th quartile. Error bars represent 1.5 times the upper or lower interquartile range. We will use horizontal brackets to indicate if there are significant differences between conditions (levels of significance: *p < 0.05; **p < 0.01; ***p < 0.001; ****p < 0.0001).

### Results

Two participants had to be excluded from the analyses because they had less than 10 valid trials in at least one condition where they followed the gaze instructions correctly. The 12 remaining participants had on average 27 valid trials per factor combination.

#### Percentage correct

**Fig. 2:**
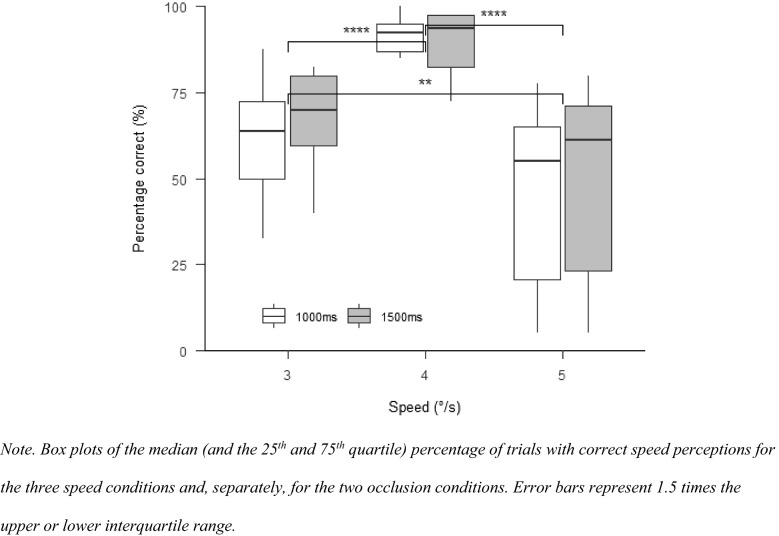
Percentage correct Experiment 1

There was a main effect for speed, $F\left( {1.91,21.00} \right) = 18.84$, $p < 0.001$, $\hat{\eta }_{{\text{p}}}^{2} = 0.631$, indicating significant differences between all speed conditions (Fig. 2) with the percentage correct being highest in the 4°/s condition and lowest in the 5°/s condition. There was neither an effect for occlusion, $F\left( {1,11} \right) = 3.43$, $p = 0.091$, $\hat{\eta }_{{\text{p}}}^{2} = 0.238$, or for the interaction between occlusion and speed, $F\left( {1.56,17.11} \right) = 1.42$, $p = 0.263$, $\hat{\eta }_{{\text{p}}}^{2} = 0.115$.

#### Saccadic response time

**Fig. 3:**
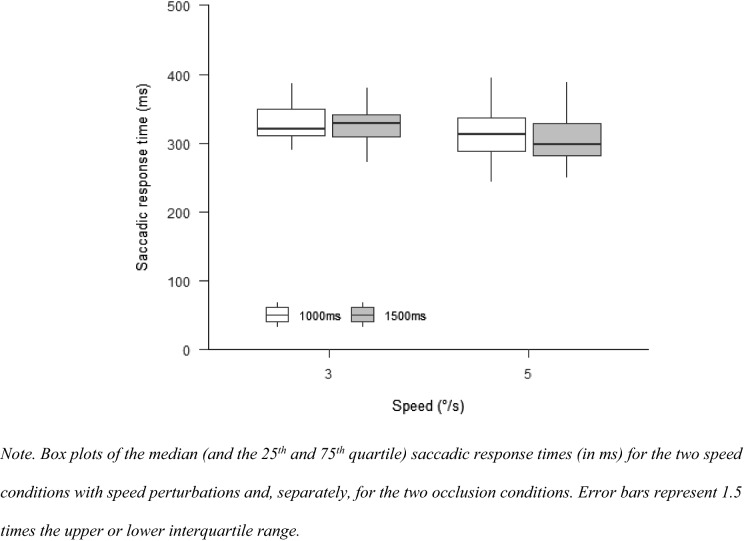
Saccadic response time Experiment 1

There was no significant difference for saccadic response times between the two speed conditions, $F\left( {1,11} \right) = 3.29$, $p = 0.097$, $\hat{\eta }_{{\text{p}}}^{2} = 0.230$, or the two occlusion conditions, $F\left( {1,11} \right) = 0.28$, $p = 0.607$, $\hat{\eta }_{{\text{p}}}^{2} = 0.025$. There was also no significant interaction between speed and occlusion time, $F\left( {1,11} \right) = 0.00$, $p = 0.988$, $\hat{\eta }_{{\text{p}}}^{2} = 0.000$ (see Fig. 3).

#### TTC prediction timing

**Fig. 4:**
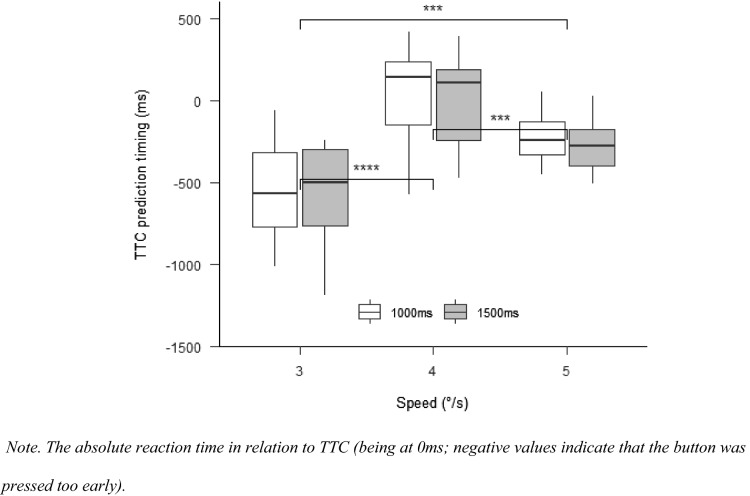
TTC prediction timing Experiment 1

There was a significant effect for speed on TTC prediction timing, $F(1.71,18.78) = 58.75$, $p < 0.001$, $\hat{\eta }_{{\text{p}}}^{2} = 0.842$ (see Fig. 4, left). Best performance was observed for the 4°/s condition, where participants continued to pursue the target object. Post hoc tests revealed that prediction timing was better in the 4°/s condition than it was in the 3°/s condition, $t\left( {11} \right) = - 13.84$, $p < 0.001$, better for the 5°/s compared with the 3°/s condition, $t\left( {11} \right) = - 5.06$, $p < 0.001$, and better for the 4°/s compared with the 5°/s condition, $t\left( {11} \right) = - 5.06$, $p < 0.001$. There was no main effect of occlusion, $F(1,11) = 0.14$, $p = 0.717$, $\hat{\eta }_{{\text{p}}}^{2 } = 0.012$, and no interaction between occlusion and speed, $F(1.69,18.56) = 3.45$, $p = 0.060$, $\hat{\eta }_{{\text{p}}}^{2} = 0.239$.

### Discussion

In Experiment 1, better performance was achieved for stimuli with a speed decrease compared to a speed increase, but best performance was observed for the 4°/s condition where there was no speed change. As expected, since speed discrimination occurred before the occlusion (i.e., before making a saccade to the + sign), there was no difference between occlusion conditions in saccadic reaction time. The prediction that participants would become better in estimating time-to-contact with more peripheral monitoring time was not supported by the results. The results also show that participants were estimating TTC with only a small error in the 4°/s condition, i.e., when they were continuing to pursue the target. With saccades and peripheral monitoring, the TTC prediction timing was better in the 5°/s compared with the 3°/s condition. Based on the saccadic response times being after around 300 ms, the peripheral monitoring time in the current experiment was 700 ms in the 1000 ms-occlusion condition and 1200 ms in the 1500 ms occlusion condition. It appears that additional monitoring does not enhance predictions beyond that possible within 700 ms. Alternatively, the information gathered during pursuit, before the saccade, might be enough to predict TTC so that peripheral monitoring does not help to improve TTC performance. To resolve this, in the next experiment we occluded the target earlier to have an occlusion condition with no peripheral monitoring time.

## Experiment 2

In Experiment 2, we shortened the available peripheral monitoring time to occlude the target 300 ms, 500 ms, 700 ms, and 900 ms after the target-speed change. Based on the saccadic response times in Experiment 1, participants should have no peripheral monitoring in the 300 ms occlusion condition. If peripheral vision is used for target monitoring, the prediction error, when the target would hit the “+”, should become lower with later target occlusions. If the prediction is made based on SPEM information alone, there should be no differences between occlusion conditions. We, again, expected better detection sensitivity in the 3°/s condition compared to the other two speed conditions, and no differences in saccadic reaction times.

### Method (changes to Experiment 1)

Compared to Experiment 1, only the occlusion times were modified (see Table 1 for the onset of target occlusions, TTC times and peripheral monitoring times). We had 30 trials for the 3°/s and 5°/s perturbation trials for each of the 4 occlusion onsets (240 trials) and 10 trials for the 4°/s no-perturbation trials in each occlusion onset condition (40 trials) leading to 280 trials in total. Since we will have the same experimental setup in Experiment 3, but with different gaze instructions, we will illustrate the eye-velocities in the three speed conditions below. We invited 14 new student participants (7 female, 7 male, age: *M* = 20.79 years).

### Results

Two participants had to be excluded from the analyses because they had less than 10 valid trials in at least one condition where they followed the gaze instructions correctly. The 12 included participants had on average 22 (out of 30) valid trials in each of the occlusion conditions for the 3°/s and 5°/s speeds and 9 valid trials (out of 10) in each of the occlusion onset conditions for the 4°/s speed.

#### Percentage correct

**Fig. 5:**
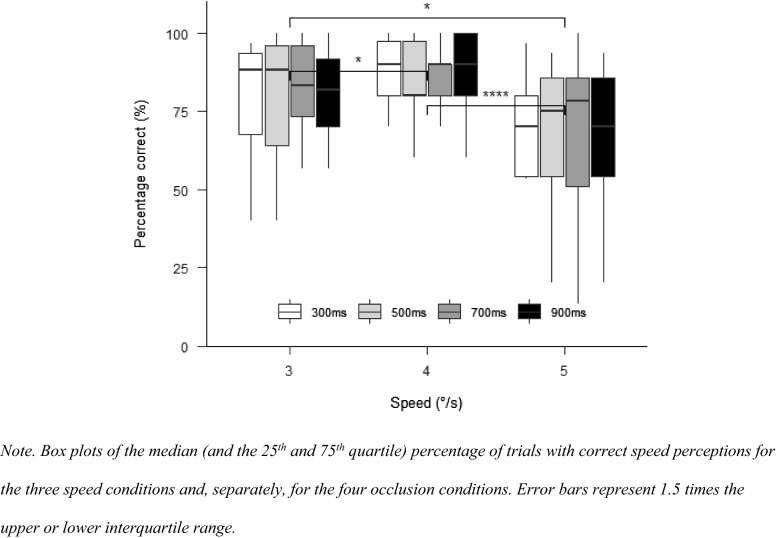
Percentage correct in Experiment 2

A main effect for speed, $F\left( {1.68,21.88} \right) = 3.81$, $p = 0.045$, $\hat{\eta }_{{\text{p}}}^{2} = 0.227$, indicated significant differences between all speed conditions (Fig. 5). Percentage correct was highest in the 4°/s condition, followed by the 3°/s condition, and then the 5°/s condition. There was neither a main effect for occlusion, $F\left( {2.90,37.70} \right) = 0.30$, $p = 0.817$, $\hat{\eta }_{{\text{p}}}^{2} = 0.023$, nor for the interaction between occlusion and speed, $F\left( {3.95,51.41} \right) = 0.31$, $p = 0.867$, $\hat{\eta }_{{\text{p}}}^{2} = 0.023$.

#### Saccadic response time

**Fig. 6:**
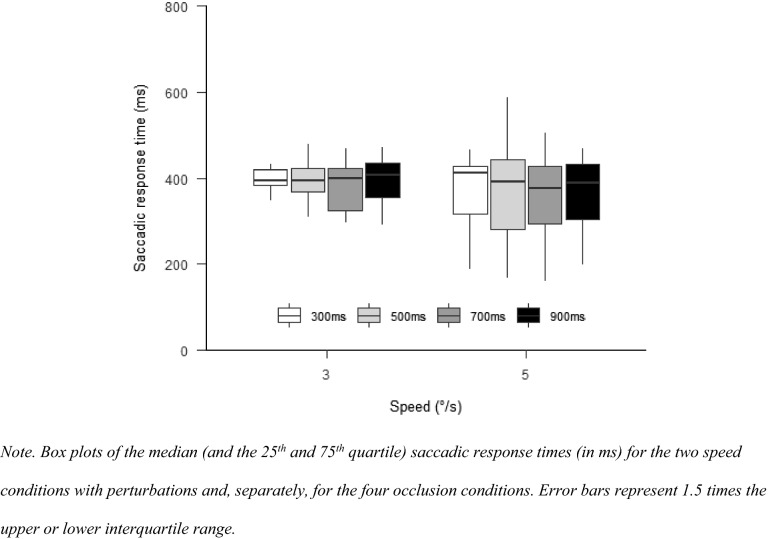
Saccadic response time Experiment 2

The saccadic response time after the perturbation did neither differ for the speed conditions, $F\left( {1,13} \right) = 0.15$, $p = 0.705$, $\hat{\eta }_{{\text{p}}}^{2} = 0.011$, nor for the different occlusion times, $F\left( {1.71,22.20} \right) = 0.80$, $p = 0.446$, $\hat{\eta }_{{\text{p}}}^{2} = 0.058$,. There was also no interaction between the two factors, $F\left( {1.88,24.39} \right) = 0.36$, $p = 0.686$, $\hat{\eta }_{{\text{p}}}^{2} = 0.027$ (Fig. 6).

#### TTC prediction timing

**Fig. 7:**
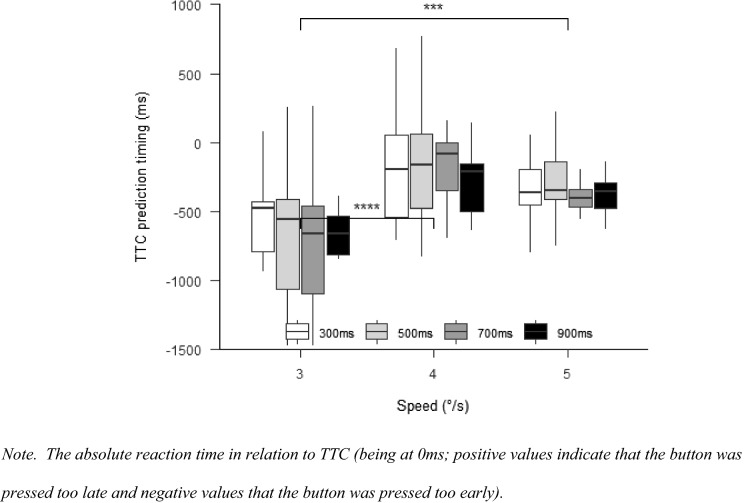
TTC prediction timing Experiment 2

There was a significant effect for speed, $F\left( {1.30,14.26} \right) = 28.00$, $p < 0.001$, $\hat{\eta }_{{\text{p}}}^{2} = 0.718$ (see Fig. 7). It can be seen that the prediction error was lower when the target did not change its speed (4°/s condition) compared to when the target speed decreased to 3°/s, $t(11) = - 10.97$, $p < 0.001$, and, when the target speed increased to 5°/s, $t(11) = 2.79$, $p = 0.003$. Prediction error was significantly lower in the 5°/s than in the 3°/s condition, $t(11) = - 3.68$, $p = 0.011$. There was no main effect of occlusion, $F\left( {2.10,23.13} \right) = 0.52$, $p = 0.608$, $\hat{\eta }_{{\text{p}}}^{2} = 0.045$, and no interaction between occlusion and speed, $F\left( {2.20,24.19} \right) = 0.80$, $p = 0.473$, $\hat{\eta }_{{\text{p}}}^{2} = 0.068$.

### Discussion

After conducting Experiment 1, it was unclear whether the predictions of time to contact were being made on the basis of pursuit and peripheral monitoring, or on the basis of pursuit alone. The predictions for Experiment 2, that best discrimination can be found for 3°/s perturbations and that saccadic reaction times should not differ between occlusion conditions, were supported by the results. Our main prediction, however, that peripheral monitoring should enable participants to become better at estimating TTC with later target occlusions, or longer peripheral monitoring times, was not supported by the results. Instead, the results from Experiment 2 extend those of Experiment 1 by strikingly suggesting that the predictions are made on the basis of the SPEMs alone instead of a combination of SPEMs, a saccade, and peripheral perception. Peripheral information does not help to improve judgements beyond what was available for the 300 ms after the change in speed. Since participants were utilizing a different gaze behavior in the 4°/s condition (pursuit) compared with the 3°/s and 5°/s condition, we conducted a final experiment, where participants were told to use SPEMs in all three conditions.

## Experiment 3

In both eye-cricket experiments, participants were asked to initiate a saccade in the 3°/s and 5°/s conditions, but to pursue the object in the 4°/s condition. To check whether the saccades impaired TTC prediction, we now compare performance in pursuit-only conditions. Instead of the saccade, we asked participants to press a button a first time when they perceived a target perturbation, but this time to continue pursuing the target with their eyes. The button should be pressed a second time when participants predicted that the target would completely align with the “+” (TTC). We expected that there should be no differences between occlusion times and between the speed conditions. Moreover, if pursuit alone is indeed better to predict TTC compared with a combination of pursuit, saccade and fixation, TTC estimations should be better in this experiment than in Experiment 2. As in previous experiments, detection sensitivity should also be better in the 3°/s condition compared with the 4°/s and 5°/s conditions. There should, however, be no differences in the reaction times for perturbation detection.

### Method (changes to Experiment 2)

The only modification in this control experiment compared to Experiment 2 is that participants should press a button when they noticed that the target changed its speed instead of initiating a saccade to the fixation “+”. The time difference between the perturbation onset and the button press was calculated (perturbation response time). Fourteen new student participants (6 female, 8 male, age: *M* = 22.20 years) were tested in this Experiment. In addition to perturbation response times, we plot the average eye-velocity for all participants for the three speed conditions and compare it to those of Experiment 2 to illustrate how eye-movements changed when the perturbation started and how the saccade affected eye-velocity. Moreover, we also calculated the relative prediction error with 0% indicating a button press at the time of speed-change onset and 100% indicating the actual TTC to compare relative TTC prediction timing in Experiments 2 and 3. We averaged the relative TTC prediction timing for each experiment and speed condition over the four occlusion conditions (because there were no differences between occlusion conditions).

### Results

Three participants had to be excluded because they had less than 10 valid trials where they followed the gaze instructions correctly. The 11 included participants had on average 25 (out of 30) valid trials in each of the occlusion × speed combinations and 9 valid trials (out of 10) in each of the occlusion conditions in the 4°/s condition.

#### Eye-velocity in Experiment 2 and 3

**Fig. 8:**
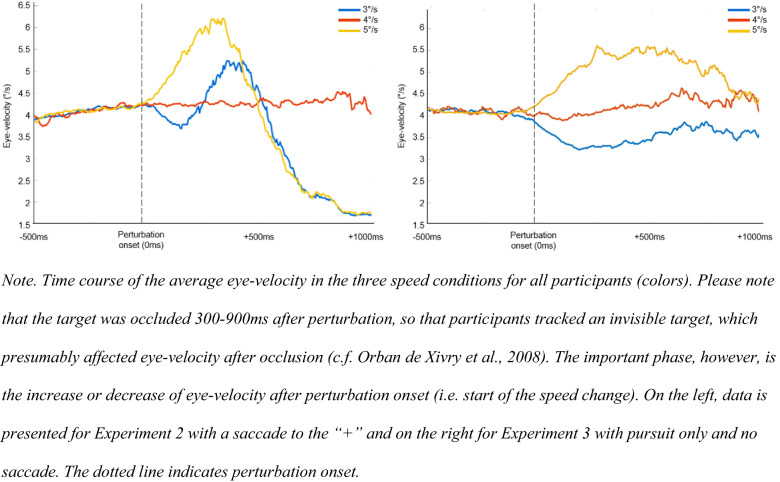
Eye-velocity in Experiment 2 and 3

The eye-velocity profiles from Experiments 2 and 3 are shown in Fig. 8. It can be seen that eye-velocity matches the target velocity (4°/s) up to the moment of perturbation onset. In both experiments, SPEM-speed followed the speed increase or decrease after the perturbation onset. In Experiment 2, a saccade was initiated when the perturbation was detected, which led to an increase followed by a decrease in eye-velocity. In contrast, in Experiment 3, participants continued to pursue the target so that eye-velocity remained at the perturbation speed.

#### Percentage correct

**Fig. 9:**
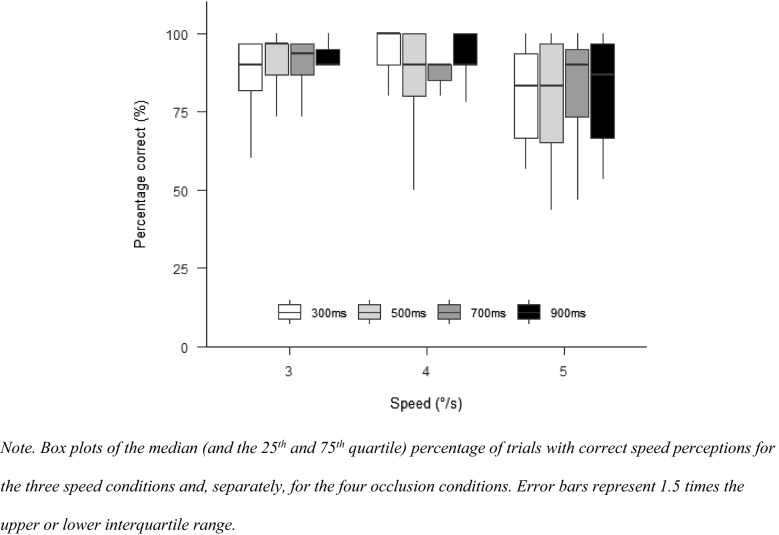
Percentage correct in Experiment 3

With SPEM in all conditions, there was no longer a significant main effect for speed, $F\left( {1.61,16.07} \right) = 2.66$, $p = 0.109$, $\hat{\eta }_{{\text{p}}}^{2} = 0.210$. There was again no effect of occlusion, $F\left( {2.24,22.45} \right) = 0.90$, $p = 0.432$, $\hat{\eta }_{{\text{p}}}^{2} = 0.082$. The interaction between speed and occlusion did also not affect the percentage correct, $F\left( {2.86,28.60} \right) = 0.52$, $p = 0.667$, $\hat{\eta }_{{\text{p}}}^{2} = 0.049$ (see Fig. 9).

#### Perturbation response time

**Fig. 10:**
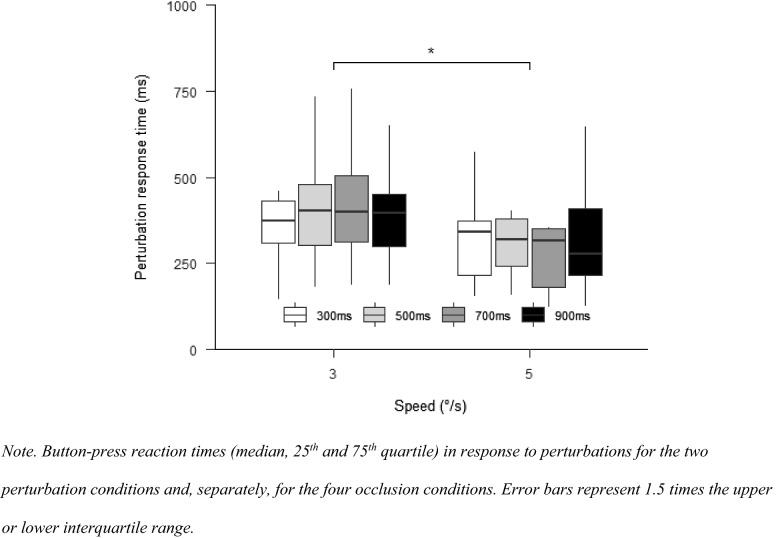
Perturbation response time in Experiment 3

The button-press reaction time differed for the speed conditions, $F(1,10) = 11.31$, $p = 0.007$, $\hat{\eta }_{{\text{p}}}^{2} = 0.531$. Faster reaction times were observed for the 5°/s than for the 3°/s condition, $t(10) = 3.36$, $p = 0.007$. There was no difference between occlusion times, $F\left( {1.40,14.00} \right) = 2.15$, $p = 0.161$, $\hat{\eta }_{{\text{p}}}^{2} = 0.177$, and there was also no interaction between the two factors, $F(2.31,23.14) = 0.41$, $p = 0.700$, $\hat{\eta }_{{\text{p}}}^{2} = 0.039$ (Fig. 10).

#### TTC prediction timing

**Fig. 11:**
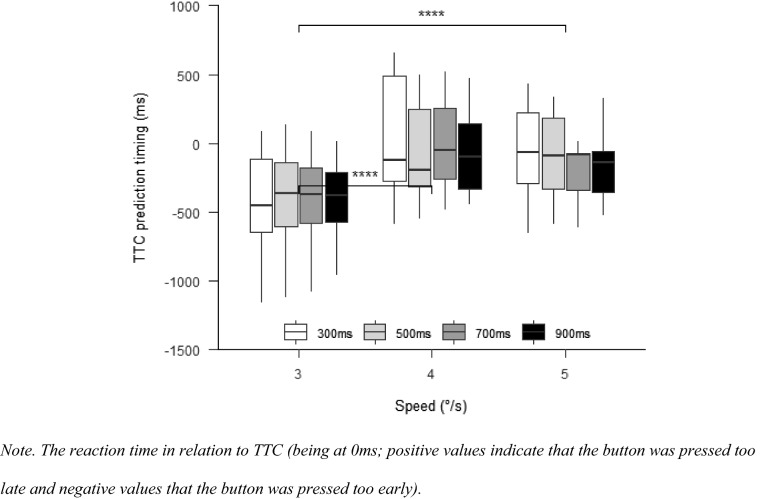
TTC prediction timing Experiment 3

There was a significant effect for speed, $F(1.84,18.42) = 34.99$, $p < 0.001$, $\hat{\eta }_{{\text{p}}}^{2} = 0.778$ (Fig. 11), with worse TTC prediction in the 3°/s condition compared to the 4°/s, $t(20) = - 8.05$, $p < 0.001$, and 5°/s conditions, $t(20) = - 6.00$, $p < 0.001$. There was no main effect for occlusion, $F(1.34,13.37) = 2.02$, $p = 0.177$, $\hat{\eta }_{{\text{p}}}^{2} = 0.168$, but an interaction between occlusion and speed, $F(3.54,35.39) = 3.86$, $p = 0.013$, $\hat{\eta }_{{\text{p}}}^{2} = 0.279$. The pairwise comparisons with Bonferroni-corrected *p* values, however, revealed no statistically significant differences between any of the occlusion conditions (all *p* > 0.266).

**Fig. 12:**
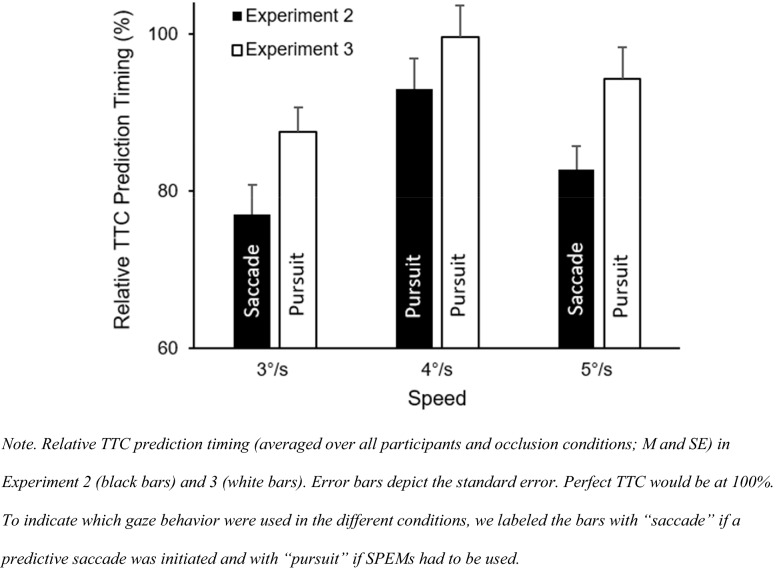
Relative TTC prediction timing in Experiment 2 and 3

When comparing TTC prediction performance in this Experiment, with pursuit only, to the previous Experiment, with an anticipatory saccade, we found that the average performance in TTC prediction timing was better in all speed conditions when using pursuit eye movements (Fig. 12). Using a speed (3°/s, 4°/s, 5°/s) × experiment (Experiment 2, Experiment 3) repeated-measures ANOVA, the expected difference between experiments with better TTC prediction timing with pursuit (Experiment 3) compared with anticipatory saccade (Experiment 2) was visible by trend, *F*(1,23) = 3.35, *p* = 0.08, $\hat{\eta }_{{\text{p}}}^{2} = 0.127$. There was a significant effect of speed, *F*(2,46) = 47.02, *p* < 0.001, $\hat{\eta }_{{\text{p}}}^{2} = 0.672$ (prediction timing differed between all speed conditions, all *p* < 0.01), but not for the interaction between speed and experiment, *F*(2,46) = 1.65, *p* = 0.21, $\hat{\eta }_{{\text{p}}}^{2} = 0.07$.

#### Exploratory results on saccadic suppression effects

**Fig. 13:**
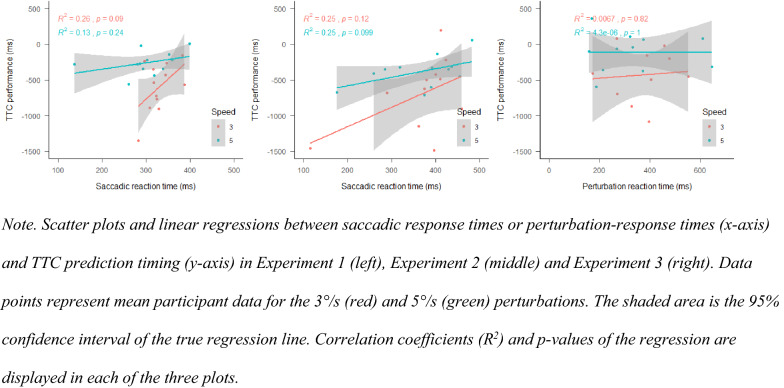
Scatter plots and linear regressions between perturbation and TTC predictions in Exp. 1, 2 and 3

It could be the case that impairments in motion perception when using peripheral vision can be explained at least in part by saccadic suppression (Binda & Morrone, 2018; Ross et al., 1997, 2001). Due to the saccade and the masking of information, crucial target speed information might have been suppressed. An indicator of the negative impact of saccades could be a correlation between saccade reaction time and TTC performance. If longer processing times alone explain better performance, later saccades should lead to better performance. Post hoc analyses revealed that this was, by trend, the case in Experiments 1 and 2 (see Fig. 13). In contrast, if information processing was not interrupted by a saccade (Experiment 3), this trend is no longer found. This could mean that, if saccades are initiated very early, the new target speed was not taken into account to predict TTC. Future research should establish whether impairments are explained by negative effects of saccades, misperceptions in peripheral vision or a combination of both.

#### Discussion

Since there were no differences in TTC performance between occlusion conditions, a short period of 300 ms after perturbation onset seems to be enough to predict TTC. The result that TTC prediction in the 300 ms was better compared to the 900 ms occlusion condition in the 4°/s and 5°/s condition underlines that only short information may be required to estimate when a target will hit another object (Fooken et al., 2016), and that further information could impair performance. As predicted, TTC performance was at least by trend better with pursuit only, compared to the previous experiment, with a combination of pursuit, saccade and a fixating ahead of the target (Fig. 12). Relative TTC performance improved on average by 9.6% when using SPEMs compared with a saccade and subsequent peripheral monitoring. It might be that using peripheral vision during SPEM to update the distance to static TTC area (“+”) is more effective for estimating TTC than using peripheral vision to update the peripherally moving target. As expected, there was no difference in discrimination performance between occlusion conditions. Contrary to our expectations, speed discrimination performance was similar in all speed conditions. The button-press response times relative to the perturbation onset were faster for target-speed increases compared to target-speed decreases, which was also not expected. Our additional exploratory analyses show that SPEMs that are not interrupted by saccades allow for a better detection performance.

## Overall discussion

In this study, we investigated whether predictive saccades facilitate the processing of information in peripheral vision when tracking a moving target. We evoked the typical eye-movements observed in cricket and other interceptive sports, starting with a SPEM, followed by a saccade, and a subsequent fixation ahead of a target. The results show that the information gathered during SPEMs is sufficient to estimate when the target will hit the fixated location, and that peripheral monitoring does not help or is not used to improve this estimation. Finally, in the last experiment (Experiment 3), we could show that it may actually be beneficial to use SPEMs rather than a saccade ahead of the target to predict its TTC with another object. Thus, taken together, results converge on the idea that predictive saccades that move fixation ahead of a target are unlikely to be performed to enhance the ability to peripherally monitor a moving target.

### Motion perception with foveal vision during SPEM vs. peripheral vision during fixation

Our results show that a target speed-change to 3°/s is better detected than target speed-change to 5°/s during peripheral viewing (Experiment 1 and 2) and that there are no differences and generally higher correct speed perceptions during SPEMs (Experiment 3). These results support a general sensitivity for detecting speed changes during SPEM (Gegenfurtner et al., 2003), particularly for detecting objects that decrease their speed (Calderone & Kaiser, 1989).

Results also show that object-speed perception is impaired with peripheral vision. TTC prediction timing was impaired when using peripheral vision after an anticipatory saccade compared to when using SPEM and foveal vision. That motion perception is better with SPEMs might be due to the additional extraretinal information (efference copy of eye-movements) which are not available during fixation (Fooken et al., 2021). Moreover, when peripheral vision was used, TTC prediction timing was more impaired when targets speed decreased compared to when it increased. One reason for lowest TTC performance for 3°/s trials could be that motion is perceived even slower in peripheral vision (Hassan et al., 2016; Traschütz et al., 2012). Thus, even if the perturbation to 3°/s was detected (saccade was initiated), it seems dysfunctional to use peripheral vision for further monitoring if the TTC is crucial for performance. One potential reason for the greater peripheral misperception is the lower density of ganglion cells found in the peripheral retina (Hassan et al., 2016). Some researchers reported that misperceptions also occur during SPEM (Aubert, 1886; Fleischl, 1882; Freeman & Banks, 1998; Freeman et al., 2010; Morvan & Wexler, 2009; Souman et al., 2005; Turano & Heidenreich, 1999). Based on our results, however, this pursuit effect seems to be smaller than what occurs peripherally during fixation.

Another, more phenomenologically driven interpretation might be related to our daily visual experience. In racquet sports, for example, it is typically the case that the ball has the highest speed shortly after it was thrown or hit by the opponent, before it subsequently decelerates on its flight to the other player (Croft et al., 2008; Müller et al., 2009). Thus, the sensitivity to perceive a target speed decreases better than an increase might be influenced by our predictions rooted in our visual expertise. It should be noted, though that sports research and basic research need to be distinguished, because in research on naïve physics, observers expect the ball to increase its speed even after it has left the hand (Hecht & Bertamini, 2000).

### Testing the peripheral monitoring hypothesis

According to Mann et al. (2013), predictive saccades could help to better track the ball after the bounce, by comparing the predicted and actual ball velocities to detect changes in the motion of the ball. Since a location close to the predicted ball-bounce point is fixated after the predictive saccade and the ball can only be viewed peripherally, a peripheral monitoring strategy could be expected. So far, it was unclear how SPEM, the predictive saccade and peripheral viewing contributed to peripheral motion-perception performance. Previous research showed that short periods of pursuit tracking allow one to predict if an object will hit a target area (Fooken et al., 2016; Spering et al., 2011). This result is supported by the current results, because participants were able to estimate TTC based on only 300 ms of visual information (i.e., the time between perturbation onset and saccade onset; see Experiments 2 and 3). Surprisingly, additional peripheral viewing time did not improve TTC predictions (nor did additional central viewing time in Experiment 3). One reason why performance did not improve with saccades and additional peripheral viewing time could be that the target perturbation was perceived (as saccades were correctly initiated) but that target velocity was not updated before the saccade was initiated. Participants might have predicted TTC based on the pre-perturbation speed (4°/s) rather than the new speed after perturbation. If this would be the case, incorrect extraretinal signals might play a role here (Fooken et al., 2021). In pursuit only trials (Experiment 3), information processing was not interrupted so that there was enough time to update motion speed information. Our findings support those by Braun et al. (2010) who found that the detection of speed changes during SPEM depends primarily on the retinal speed of the motion but that there is also an asymmetry whether the retinal speed is caused by pursuit (as in our Experiment 3) or object motion (as in our Experiments 1 and 2) with higher sensitivity for SPEM-induced retinal motion. Figure 12 shows that TTC prediction was better when using pursuit only. Such an interpretation seems to be in line with the finding by Gegenfurtner et al. (2003), who showed that psychometric (perturbation detection accuracy) and oculometric (accuracy of adjusting eye-movement speed to perturbations) performanc are not correlated. In the current study, perturbations could have been detected (correct eye-movement), but the speed was maybe not perceived correctly (incorrect perception).

### Limitations and future directions

In this task, we have presented a very simplified version of an interceptive (cricket) task because the tested velocities are much slower than in cricket and the ball did not bounce. Replicating the current findings with greater target speeds, more natural viewing angles, and motor responses seems warranted. Therefore, we aim to conduct a study with a virtual-reality situation, where ball trajectories can be perfectly replicated and ball speeds and viewing angles can be manipulated. Additionally, for realistic motor responses, the ball should be ‘hit’ with a real racquet with movement behavior captured with movement analyses systems (e.g., VICON or Optitrack).

Future studies could explore if a velocity memory of the predictive saccade informs the motion prediction as proposed by Makin and Poliakoff (2011) and Makin (2018). This might help to better understand how target movements are extrapolated even if the target gets occluded or, as in our case, seems no longer be updated after the pursuit phase. It might also reveal how the oculomotor or the perception “system” is involved in the processing of target position signals.
